# Neural Correlates of Belief and Emotion Attribution in Schizophrenia

**DOI:** 10.1371/journal.pone.0165546

**Published:** 2016-11-03

**Authors:** Junghee Lee, William P. Horan, Jonathan K. Wynn, Michael F. Green

**Affiliations:** 1 Semel Institute for Neuroscience and Human Behavior, University of California Los Angeles, Los Angeles, California, United States of America; 2 VA Greater Los Angeles Healthcare System, Los Angeles, California, United States of America; University of Pennsylvania Perelman School of Medicine, UNITED STATES

## Abstract

Impaired mental state attribution is a core social cognitive deficit in schizophrenia. With functional magnetic resonance imaging (fMRI), this study examined the extent to which the core neural system of mental state attribution is involved in mental state attribution, focusing on belief attribution and emotion attribution. Fifteen schizophrenia outpatients and 14 healthy controls performed two mental state attribution tasks in the scanner. In a Belief Attribution Task, after reading a short vignette, participants were asked infer either the belief of a character (a false belief condition) or a physical state of an affair (a false photograph condition). In an Emotion Attribution Task, participants were asked either to judge whether character(s) in pictures felt unpleasant, pleasant, or neutral emotion (other condition) or to look at pictures that did not have any human characters (view condition). fMRI data were analyzing focusing on a priori regions of interest (ROIs) of the core neural systems of mental state attribution: the medial prefrontal cortex (mPFC), temporoparietal junction (TPJ) and precuneus. An exploratory whole brain analysis was also performed. Both patients and controls showed greater activation in all four ROIs during the Belief Attribution Task than the Emotion Attribution Task. Patients also showed less activation in the precuneus and left TPJ compared to controls during the Belief Attribution Task. No significant group difference was found during the Emotion Attribution Task in any of ROIs. An exploratory whole brain analysis showed a similar pattern of neural activations. These findings suggest that while schizophrenia patients rely on the same neural network as controls do when attributing beliefs of others, patients did not show reduced activation in the key regions such as the TPJ. Further, this study did not find evidence for aberrant neural activation during emotion attribution or recruitment of compensatory brain regions in schizophrenia.

## Introduction

Assessing how others feel or what others think is crucial to understanding and responding appropriately to their behaviors in everyday life. This ability to infer the emotion, beliefs and intentions of others is referred to as mental state attribution [1, 2]. Schizophrenia patients show impaired performance across diverse mental state attribution tasks [3], and the field is beginning to understand neural mechanisms of this impairment [4]. To provide further information on the neural correlates of impaired mental state attribution in schizophrenia, this study examined the extent to which the core neural mechanism of mental state attribution is involved indifferent types of mental state attribution in schizophrenia, focusing on belief and emotion attribution.

Belief attribution involves an ability to infer beliefs or thoughts of others (e.g., he is going back to his office after work because he thinks he left his wallet there), whereas emotion attribution involves an ability to attribute emotional states to others (e.g., she is crying—I think she is sad after watching the movie “Steel Magnolias.”). Emotion attribution is sometimes referred to as cognitive empathy. A set of brain regions, including the medial prefrontal cortex, temporoparietal junction (TPJ) and the precuneus, have consistently shown to be activated in both belief and emotion attribution tasks [4–8] and are considered the core neural mechanisms of the mental state attribution system. In addition to these core regions, belief and emotion attribution are also associated with distinct neural regions [5–8]. Specifically, the posterior cingulate gyrus and amygdala are more associated with emotion attribution; the superior frontal regions and the middle frontal gyrus were more related to belief attribution. These findings suggest that belief attribution and emotion attribution involves some overlapping and some distinct neural regions.

A large body of research using behavioral paradigms has shown that individuals with schizophrenia have deficits in both belief and emotion attribution. For example, compared to healthy controls, patients have difficulty inferring thoughts or intention of others [9]; patients are also less accurate at detecting lies or sarcasm of others when watching two people interacting in a video clip [10–12]. Similarly, patients have difficulty attributing emotional states to another person, as seen in a variety of paradigms [13, 14]. Further, schizophrenia patients show similar levels of impairment across belief and emotion attribution tasks, as evidenced by a meta-analysis [15]: an effect size of patient-control difference on a commonly used emotion attribution task (i.e., reading the mind in the eyes) was 0.90 and an effect size on a commonly used test of belief attribution (i.e., false-belief task) was 1.06.

These relatively similar levels of impairment across belief and emotion attribution in schizophrenia raise a question as to whether both impairments stem from the same neural regions of the core component of the mental state attribution system. Functional neuroimaging studies of belief attribution in schizophrenia have shown reduced neural activation in several brain regions, including the TPJ, precuneus and medial prefrontal cortex [16–19]. It should be noted that the TPJ, precuneus and medial prefrontal cortex are considered as a core mentalizing system because they have consistently been activated in various mentalizing tasks [20, 21]. The role of the core system in emotion attribution in schizophrenia is less clear. Some studies on emotion attribution in schizophrenia have shown reduced activation in the superior temporal gyrus [22, 23] and precuneus [24], whereas others implicated abnormalities in different areas, such as hyperactivation in the insula [25]. Thus, it remains unclear whether both impairments involve abnormal neural activations of the core system in schizophrenia.

This study aimed to examine the extent to which neural activation in the core neural system of mental state attribution was aberrant in impaired belief and emotion attribution in schizophrenia. To do so, we administered a Belief Attribution Task and an Emotion Attribution Task in the same functional MRI (fMRI) session. The Belief Attribution Task employed false belief stories, in which participants are asked to infer the beliefs of another person even when these beliefs differs from the true state of affairs. In the Emotion Attribution Task, we employed visual images of people and asked participants to infer the affective state of another person. We hypothesized that the core neural system of mental state attribution would show reduced activation in schizophrenia patients compared to healthy controls during both the Belief Attribution Task and Emotion Attribution Task in a comparable way. This hypothesis was tested by examining the levels of neural activation of schizophrenia patients and healthy controls during two tasks in *a priori* regions of interest (ROIs) of the core system: the medial prefrontal cortex, TPJ and precuneus. These ROIS are selected based on a recent review paper in neural correlates of social cognitive impairment in schizophrenia [4]. In addition to these *a priori* ROIs, we also conducted an exploratory whole brain analyses to examine neural activation associated with belief and emotion attribution outside these ROIs.

## Method

### Participants

Seventeen patients with schizophrenia and 15 healthy controls participated in this study. Schizophrenia patients were recruited from outpatient clinics at University of California, Los Angeles (UCLA) and the Veterans Affairs Greater Los Angeles Healthcare System (GLA) and from local board and care facilities in Los Angeles. Healthy controls were recruited through flyers distributed in the local community and website postings. Patients were included if they had: 1) a diagnosis of schizophrenia based on the Structured Clinical Interview for DSM-IV Axis I Disorders (SCID) [26]; 2) no substance abuse in the past month or dependence in the last six months; and 3) IQ not less than 70 based on review of medical records. All patients were medicated and clinically stable at the time of testing (e.g., no change in psychoactive medication in the 6 weeks prior to study participation, no inpatient hospitalization for 3 months prior to study participation). Two patients were taking typical antipsychotic medication, 1 patient was taking both typical and atypical antipsychotic medication and 9 patients were taking atypical antipsychotic medication. Healthy controls were excluded if they had: 1) history of schizophrenia or psychotic disorder, bipolar disorder, recurrent depression, substance dependence, or any substance abuse in the past month based on the SCID [26]; 2) any of the following Axis II disorders: avoidant, paranoid, schizoid or schizotypal based on the SCID for Axis II disorders [27]; 3) schizophrenia or other psychotic disorder in a first-degree relative. Additional selection criteria for both patients and healthy controls were: 1) no history of loss of consciousness for more than one hour; 2) no identifiable neurological disorder; and 3) sufficient fluency in English to understand testing procedures.

All SCID interviewers were trained to a minimum kappa of 0.75 for key psychotic and mood items through the Treatment Unit of the VA VISN 22 Mental Illness Research, Education, and Clinical Center (MIRECC). All participants were evaluated for the capacity to give informed consent and provided written informed consent after all procedures were fully explained, according to procedures approved by the Institutional Review Boards at UCLA and GLA.

### Procedures

Participants performed the Belief Attribution Task and the Emotion Attribution Task in the scanner using MR-compatible LCD goggles (Resonance Technology, Northridge, CA). The order of tasks was counterbalanced across participants to control for any potential effect of scanner baseline drift that could be confounding the comparison of neural activation differences between two tasks.

#### Belief Attribution Task

The Belief Attribution Task, adapted from Saxe and Kanwisher [28], employed an event-related design in which each trial consisted of a vignette and a two-alternative fill-in-the-blank question. There were three conditions, each with 12 vignettes: a false belief condition, a false photograph condition, and a simple reading condition. The false belief condition included vignettes with two characters and asked subjects to infer the beliefs of a character even when these beliefs were different from the actual state of affairs. The false photograph condition included vignettes describing a photograph or painting that differed from the actual state of affairs. The simple reading condition included stories describing non-human objects.

The Belief Attribution Task consisted of 6 runs, with 6 trials per run (2 trials of each condition). At the onset of each trial, a vignette was presented for 12 seconds; then, a fill-in-the-blank question was presented for 10 seconds while the vignette was still visible. The fill-in-the-blank question consisted of a single sentence with a word missing, presented above two alternatives. After the vignette and question disappeared, a probe was presented for 3 s, prompting subjects to respond by pressing the corresponding button. The intertrial intervals (ITIs) were jittered between 12 and 18 seconds.

#### Emotion Attribution Task

The Emotion Attribution Task, modeled after Ochsner et al. [29], used a block design with images from the International Affective Picture Series (IAPS, [30]) and had 3 conditions: an other-attribution condition, a view condition, and a subjective-labeling condition. For both the other-attribution condition and subjective-labeling condition, participants viewed 8 images from each of the three categories: aversive (mean valence rating = 2.7, mean arousal rating = 4.7), pleasant (mean valence rating = 7.5, mean arousal rating = 4.7), and neutral (mean valence rating = 5.0, mean arousal rating = 3.1). All images depicted social situations with human figures (either one or multiple characters). For the other-attribution condition, participants were asked to judge whether the character(s) in the picture felt a pleasant, unpleasant, or neutral emotion by pressing the corresponding button. For the view condition, 24 neutral images without human figures were used. In the view condition, participants were asked to look at the image without making any judgment. This condition as a perceptual baseline condition was designed to control for processing visual stimuli. For the subjective-labeling condition, participants were asked to indicate how they felt (pleasant, unpleasant, or neutral) while viewing each picture. Data from this condition was not part of this study that focused on the attribution of others’ mental states although the condition was included as a non-interest, nuisance variable in the data analysis.

The task involved four runs, each with two blocks of the other-attribution condition, two blocks of the subjective-labeling condition and four blocks of the view condition. Each block consisted of 3 trials, in which each trial consisted of a 2-sec instruction (e.g., other) followed by an image display for 4 seconds. In the other condition, subjects had 3.5 seconds to make a response after each trial; and in the view condition, they saw a gray screen for 3.5 seconds.

### Imaging Data Acquisition and Analysis

Imaging data were collected using a 3T scanner (Siemens Trio, Erlangen, Germany) located in the UCLA Ahmanson Lovelace Brain Mapping Center. For anatomical reference, a high-resolution echo planar axial T2-weighted series was obtained for each subject prior to functional scanning (TR = 5000 ms, TE = 30 ms, flip angle = 90 degrees, 33 slices, FOV 22 cm). A T2*-weighted gradient-echo sequence was used to detect blood-oxygen level-dependent (BOLD) signal (TR = 2000 ms, TE = 30 ms, flip angle = 75 degrees, voxel size of 3.4 x 3.4 x 4.00 mm), acquiring 33 slices parallel to the AC-PC plane. The fMRI data from the Belief Attribution Task on a subset of participants were previously reported [16]

fMRI data were analyzed using FMRI Software Library (FSL) [31]. The pre-statistics processing included motion correction using MCFLIRT (Motion Correction using FMRIB’s Linear Imaging Registration Tool) [32], non-brain removal using BET (Brain Extraction Tool) [33], spatial smoothing using a Gaussian kernel of the full width at half maximum 5 mm, grand-mean intensity normalization by a single multiplicative factor, and high pass temporal filtering (Gaussian weighted LSF straight line fitting with sigma = 50.0 s). To ensure that any potential group differences were not likely to be explained by any motion artifacts, we applied the following procedures. First, we visually inspected all the data for obvious motion artifacts and observed that no subject showed movement greater than 2mm during scans. Second, we examined whether there was any group difference in head motion with MCFLIRT and found that both groups showed comparable head motion during the scan (Belief Attribution Task, patients = .12mm (SD = .06) and controls = .10mm (SD = .04); Emotion Attribution Task, patients = .10mm (SD = .04) and controls = .12mm (SD = .06)). Finally, we included head motion parameters as confound explanatory regressors in the first-level analyses to remove any residual effects of motion that could influence results after motion correction. To facilitate multi-subject analyses, statistical images created for each participant were normalized into a standard space from the Montreal Neurological Institute (MNI) using affine transformation with FLIRT (FMRIB’s Linear Image Registration Tool) [34].

The first-level analyses of fMRI data from each task were conducted for each run of each participant. For both tasks, task regressors were created by convolving condition events with a canonical hemodynamic response function. For the Emotion Attribution Task, 3 task conditions were modeled but the subjective-labeling condition (that was not part of this study) was served as a regressor of no-interest. The main contrast of interest for the Emotion Attribution Task was: other attribution vs. view conditions. Similarly, data from all 3 conditions of the Belief Attribution Task were also modeled. The main contrast of interest for the Belief Attribution Task was: false belief vs. false photograph conditions. For second-level analysis for each task, we analyzed the data across runs for each subject using a fixed effects model in FLAME (FRMIB’s Local Analysis of Mixed Effects) [35, 36].

**Fig 1:**
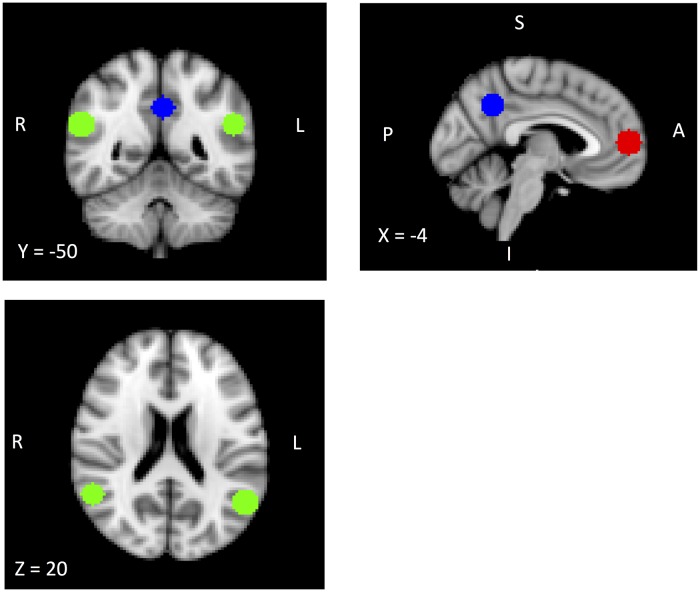
A priori regions of interests (ROIs). The bilateral TPJ is depicted in green (left TPJ, X = -52, Y = -56, Z = 24; right TPJ, X = 56, Y = -54, Z = 24). The mPFC is depicted in red (X = -4, Y = 56, Z = 8). The precuneus is depicted in blue (X = -2, Y = -56, Z = 36). Coordinates are given in Montreal Neurological Institute (MNI) space.

For ROI analyses, we created 4 spherical ROIs (10 mm diameters) centered around peak coordinates for the medial prefrontal cortex (mPFC), bilateral TPJ and precuneus that were identified in a meta-analysis of previous fMRI studies on mental state attribution [37]. Fig 1 displays ROIs with corresponding coordinates. Then, for each ROI, we extracted mean-beta values for the main contrast of interest of each task and conducted a repeated measures ANOVA with task as within-subject factor and group as between-subject factor in each ROI. For each ROI, we also examined the association between mean-beta values and performance on each task within each group using Spearman’s rho.

We also conducted an exploratory whole brain analysis to examine whether groups showed differential activation outside these four ROIs during the Belief Attribution Task and Emotion Attribution Task. Specifically, for each task, a mixed-effects model (FLAME stage 1+2 of FSL) [35, 36] was performed with behavioral performance as a covariate, to characterize neural activation in each group separately and to directly compare patients to controls for each contrast of interest. All statistical images for the whole brain analysis were thresholded using a z value > 2.3 with a cluster probability of p = 0.05, corrected for multiple comparison using Gaussian random field theory [38].

## Results

Two patients were excluded from analyses due to below-chance level performance and one control was excluded due to technical difficulty. Therefore, 15 schizophrenia patients and 14 healthy controls were included in the following analyses. There was no statistically significant difference on age, parental education and personal education between schizophrenia patients and controls (see Table 1).

**Table 1:** Demographic information and behavioral performance of schizophrenia patients and controls †.

	SZ (N = 15)	HC (N = 14)
Age	38.4 (10.3)	41.8 (7.4)
Personal education (yrs.)	12.8 (2.5)	14.3 (1.3)
Parental education (yrs.)	13.4 (3.0)	14.2 (2.8)
Belief Attribution Task		
False Belief	8.6 (1.3)	10.3 (1.2)
False Photograph	7.5 (1.7)	9.1 (1.2)
Simple Reading	9.8 (1.4)	11.2 (.8)
Emotion Attribution Task		
Other-positive	2.7 (.4)	2.9 (.03)
Other-negative	1.4 (.3)	1.2 (.1)
Other-neutral	2.1 (.3)	1.9 (.2)

^†^ Values are given as mean (standard deviation).

### Behavioral performance

Table 1 shows behavioral performance of patients and controls. For the Belief Attribution Task, a 3 (condition) by 2 (group) repeated measures ANOVA showed a significant effect of condition (F_2,54_ = 34.31, p < .01, η_p_^2^ = .56) and a significant effect of group (F_1,27_ = 15.02, p < .001, η_p_^2^ = .36). The condition by group interaction was not significant. Both groups performed best on the simple reading condition and performed worse on the false photograph condition (simple reading < false belief, p < .01; and false belief < false photograph, p < .001). Although the group difference was significant, the patients performed relatively well across conditions (see Table 1). For the Emotion Attribution Task, a 3 (condition) by 2 (group) repeated measures ANVOA found a significant effect of condition (F_2,56_ = 211.61, p < .001, η_p_^2^ = .88) and a significant group by condition effect (F_2,56_ = 5.64, p < .01, η_p_^2^ = .17), but no significant group effect. Both groups rated positive images as pleasant and aversive images as unpleasant (aversive < neutral, p < .001; and neutral < positive, p < .001), but compared to controls, schizophrenia patients rated positive images less pleasant (p < .05) and aversive images as more unpleasant (p < .05).

### ROI analysis

**Fig 2:**
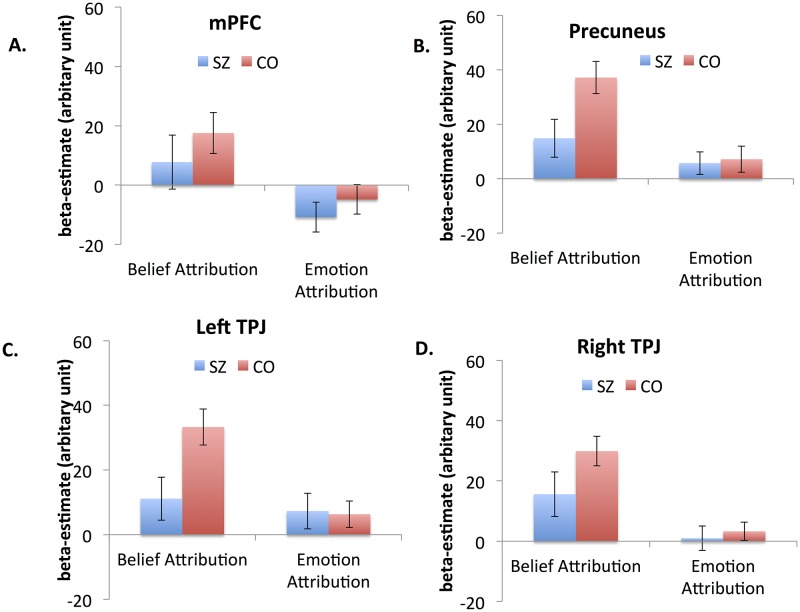
Beta values of the mPFC (A), precuneus (B), left TPJ (C) and right TPJ (D) during the Belief Attribution Task and Emotion Attribution Task. Values are given as mean (standard error).

Fig 2 shows the levels of neural activation of the two groups in each ROI during the Belief Attribution Task and Emotion Attribution Task. For the mPFC, a repeated meausres ANOVA showed a significant effect of task (F_1,27_ = 12.52, p < .01, η_p_^2^ = .31) but no other significant effect. Both schizophrenia patients and controls showed greater activation during the Belief Attribution Task than the Emotion Attribuiton Task. In the Precuneus, there was a significant effect of task (F_1,27_ = 12.01, p < .01, η_p_^2^ = .31). Both a task by group interaction (F_1,27_ = 3.29, p = .08, η_p_^2^ = .11) and a main effect of group (F_1,27_ = 3.71, p = .06, η_p_^2^ = .12) approached significance. Both schizophrenia patients and controls showed greater activation during the Belief Attribution Task. Schizophrenia patients showed somewhat reduced activation compared to controls and this pattern was present during the Belief Attribution Task (p < .05), but not during the Emotion Attribution Task (p < .8).

In the left TPJ, there were a significant effect of task (F_1,27_ = 9.17, p < .01, η_p_^2^ = .26) and a marginally significant task by group interaction (F_1,27_ = 4.06, p = .054, η_p_^2^ = .13). Both schizophrenia patients and controls showed greater activation during the Belief Attribution Task compared to the Emotion Attribution Task. However, schizophrenia patients showed lower activation than controls during the Belief Attribution Task (p < .05), but not during the Emotion Attribution Task (p>.8). In the right TPJ, there was a significant effect of task (F_1,27_ = 21.86, p < .001, η_p_^2^ = .44) but no other effect was significant. Similar to other ROIs, both groups showed greater activation during the Belief Attribution Task. Finally, when examining the association between mean-beta values of each ROI and performance on each task, no significant association after correcting for multiple comparisons was observed in either group.

### Exploratory whole brain analysis

**Fig 3:**
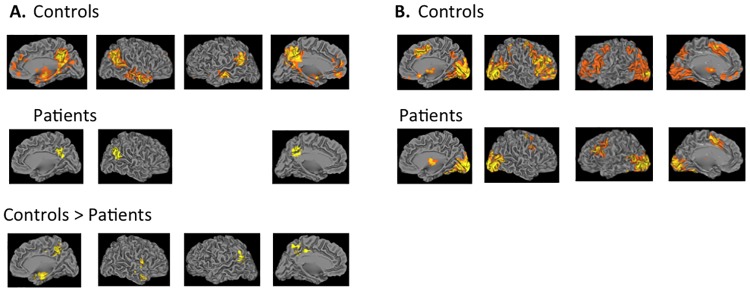
Brain activation patterns of the whole brain analysis. 3A shows neural activation patterns for the contrast of false belief > false photograph condition of the Belief Attribution Task in controls, patients and controls > patients. There was no area in which patients showed significantly greater activation than controls. 3B shows neural activation patterns for the contrast of other attribution > viewing condition in controls and patients. Direct group comparison did not show any brain regions with significantly different activation between groups. All statistical images were thresholded using a z value > 2.3 with a corrected cluster probability of p = 0.05 to control for multiple comparisons using Gaussian random field theory.

Fig 3 and Table 2 show brain regions that showed significant activation above threshold in the whole brain analysis and their corresponding coordinates. During the false belief versus false photograph conditions of the Belief Attribution Task (Fig 3A), controls showed increased activation in the several brain regions including the precuneus, bilateral TPJ, bilateral middle temporal gyrus, mPFC, the occipital cortex and putamen whereas patients showed increased activations in the right TPJ and precuenus. Direct group comparison showed greater activation of controls in the left TPJ and precuneus. Patients did not show greater activation than controls in any regions. During the other attribution versus viewing conditions of the Emotion Attribution Task (Fig 3B), controls showed increased activation in several brain regions including the bilateral lateral prefrontal cortex, the occipital cortex, dorsomedial prefrontal cortex, and precuneus. Patients showed greater activation in a smaller set of brain regions including the left lateral prefrontal cortex, occipital cortex, and supplementary motor cortex. In a direct comparison, no significant difference between the groups was found in any regions

**Table 2:** Locations of significant activation for the Belief Attribution Task and Emotion Attribution Task.

Cluster	Voxels	Z value	X^a^	Y	Z	Label
**Belief Attribution Task**						
Controls						
1	4903	6.87	6	-48	42	Precuneus extending to the posterior cingulate gyrus
2	1764	5.81	46	-56	30	Temporoparietal junction extending to the angular gyrus
3	1426	5.48	-44	-58	26	Temporoparietal junction extending to the angular gyrus
4	1143	5.07	50	4	-32	Middle temporal gyrus
5	509	4.79	-62	-18	-10	Middle temporal gyrus
6	414	4.37	-28	0	-18	Parahippocampal gyrus extending to the hippocampus and amygdala
7	410	3.73	12	58	6	Anterior cingulate gyrus extending to the medial prefrontal cortex and frontal pole
8	318	4.19	-8	46	-12	Medial prefrontal cortex
Patients						
1	494	3.47	4	-56	26	Precuneus extending to the posterior cingulate gyrus
2	344	3.51	56	-52	20	Temporoparietal junction extending to the angular gyrus
Controls > Patients						
1	800	3.84	34	4	-20	Temporal pole extending to the paraphippocampal gyrus
2	796	3.81	4	-42	52	Precuneus extending to the posterior cingulate cortex
3	417	3.56	-40	-56	22	Temporoparietal junction extending to the angular gyrus
**Emotion Attribution Task**						
Controls						
1	15775	9.44	-26	-96	0	Occipital cortex extending to the lateral occipital cortex
2	6897	6.67	-4	12	50	Anterior cingulate gyrus extending to the paracingulate gyrus, left middle- and inferior frontal gyrus and insular cortex
3	4003	5.19	-40	2	58	Middle frontal gyrus extending to inferior frontal gyrus and insular cortex
4	1127	4.86	-12	-4	-6	Basal ganglia including thalamus and putaman
5	670	4.83	42	-44	42	Supramarginal gyrs extending to the superior parietal lobule
Patients						
1	12825	6.15	-40	-84	-12	Occipital cortex extending to the lateral occipital cortex
2	1315	4.16	-34	6	28	Precentral gyrus extending to the middle frontal gyrus
3	716	3.68	8	-26	-6	Basal ganglia including thamamus and putaman
4	708	4	30	-4	48	Precentral gyrus extending to the middle frontal gyrus
5	442	4.27	-10	12	46	Anterior cingulate gyrus extending to paracingulate gyrus and supplementary motor cortex

^a^ Coordinates are given in Montreal Neurological Institute (MNI) space.

## Discussion

This study examined the extent to which impaired mental state attribution in schizophrenia is associated with abnormal activation in the core neural system of mental state attribution: mPFC, TPJ and precuneus. By focusing on a priori ROIs, this study found that both schizophrenia patients and controls showed greater activation in all four ROIs during the Belief Attribution Task compared to the Emotion Attribution Task. Further, patients showed less neural activation than controls in the precuneus and left TPJ only during the Belief Attribution Task. An exploratory whole brain analysis showed a similar pattern of results. During the Belief Attribution Task, although both patients and controls showed increased activation in several areas of the core system, controls had significantly greater activation in the left TPJ and precuneus than patients. During the Emotion Attribution task, both patients and controls showed comparable activation in a large set of brain regions in a comparable way, but these areas did not overlap with *a priori* ROIs.

Previous studies examined belief attribution and emotion attribution in schizophrenia separately, and found evidence of abnormal neural activation for both types of tasks [16–19, 22, 23]. However, it was not clear to what extent belief and emotion attribution impairment relies on the core neural system of mental state attribution. By employing both the Belief Attribution Task and the Emotion Attribution Task in the same study and focusing on *a priori* ROIs, this study showed that reduced activation in the core neural system of mental state attribution is closely related to impaired belief attribution in schizophrenia. However, we did not find any evidence of a close relationship between the core neural system of mental state attribution and emotion attribution in schizophrenia. Both patients and controls activated the core system much less during emotion attribution than belief attribution and the levels of activation during the emotion attribution did not differ between patients and controls. It is unclear whether the core neural system is more reliably associated with belief attribution in schizophrenia or is less activate during emotion attribution because the task was less difficult.

During belief attribution, patients showed reduced activation in left TP and precuneus as well as the angular gyrus compared to controls. Although the angular gyrus is not typically considered to be a core region for mentalizing, some studies have found increased activation in this region during mental state attribution tasks, so it may be a secondary component of the mentalizing network [39, 40]. Alternatively, increased activation in the angular gyrus may reflect non-social demands that are necessary to perform the activation tasks. The angular gyrus is known to be involved in multiple cognitive functions, including semantic processing and spatial attention [41]. The Belief Attribution Task in this study relied heavily on story comprehension, which could explain why patients showed reduced activation in the angular gyrus than controls.

This study has some limitations. It had a relatively small number of subjects so it was not powered to detect subtle group differences. Schizophrenia patients are also clinically stable, chronic patients and it is unclear whether a similar pattern could be observed in patients with recent-onset psychosis and whether patients with different symptom profiles may show different patterns of neural activation. The Belief Attribution Task and the Emotion Attribution Task involved stimuli presented in different modalities (verbal for belief vs. visual for emotion). It needs to be determined to what extent the use of different modalities across the tasks might have affected unique neural activation patterns of each paradigm. Additionally, whereas the false photograph condition in the Belief Attribution Task enabled us to examine neural activation of belief attribution controlling for a general reasoning ability, the Emotion Attribution Task only had a perceptual baseline condition to control for processing complex visual stimuli.

In summary, although both patients and controls showed significant neural activation in *a priori* ROIs (i.e., mPFC, TPJ and preceunus) during the Belief Attribution Task, patients showed significantly lower activation in the precuneus and left TPJ than controls. In contrast, this study did not find evidence of aberrant neural activation during emotion attribution in schizophrenia. These findings suggest that while schizophrenia patients rely on the same neural network as controls do when attributing beliefs of others instead of recruiting additional compensatory regions, they did show reduced activation in the key regions, such as the TPJ.

## Supporting Information

S1 Dataset Data file for ROI analyses are included. (SAV)
